# A Bureau of Social Hygiene

**Published:** 1913-02

**Authors:** 


### A Bureau of Social Hygiene.

The State Reformatory of New York has recently established a bureau of social hygiene, where the problems dealing with prostitution can be studied scientifically. One of the dismal failures of sociology in the past has been the failure to regulate prostitution and sexual vice. The question has been heretofore entirely one of sentiment, failing to take into account the principles on which every condition must of necessity be founded. A thorough understanding of eugenics would furnish this basis.

